# Attitudes and Exposure to Illicit Tobacco in England, 2022

**DOI:** 10.1093/ntr/ntae118

**Published:** 2024-05-16

**Authors:** Nathan Davies, Tessa Langley, Leah Jayes, Manpreet Bains, Jamie Brown, Deborah Arnott, Ilze Bogdanovica

**Affiliations:** School of Medicine, Nottingham City Hospital, University of Nottingham, Nottingham, UK; School of Medicine, Nottingham City Hospital, University of Nottingham, Nottingham, UK; SPECTRUM Consortium, Edinburgh, UK; Institute of Health and Allied Professions, Nottingham Trent University, Nottingham, UK; School of Medicine, Nottingham City Hospital, University of Nottingham, Nottingham, UK; Institute of Epidemiology & Health Care, University College London, London, UK; Action on Smoking and Health, The Foundry, London, UK; School of Medicine, Nottingham City Hospital, University of Nottingham, Nottingham, UK; SPECTRUM Consortium, Edinburgh, UK

## Abstract

**Introduction:**

The United Kingdom has achieved reductions in illicit tobacco (IT) market size and share. However, there remains a 17.7% tobacco duty gap, contributing to health inequalities. In January 2024, the UK government announced a new strategy to control IT, along with provision of new funding.

**Methods:**

A representative cross-sectional survey of adults in England ran in April 2022 to evaluate attitudes and exposure to IT. Tobacco smokers were asked questions about encounters with IT, while all participants answered questions on knowledge and perspectives on IT.

**Results:**

Of 262 tobacco smokers, 18.3% (95% CI 13.8% to 23.6%) had come across IT in the past year. Men had four times the odds of encountering IT recently than women. Among 1767 adults responding to questions on IT, two-thirds agreed IT harmed children, and more than half agreed IT was linked to organized crime. Younger adults, smokers, and those in lower socioeconomic groups were less likely to agree IT was harmful.

**Conclusions:**

Exposure to IT, especially among younger males, remains a concern. While most of the public acknowledge its harm, this is not universal, and some population groups are less likely to do so.

**Implications:**

The study highlights persistent exposure to IT in England, especially among younger males, and varying perceptions of IT harm across socioeconomic groups. Tackling IT requires collaboration between health and enforcement agencies, independent of the tobacco industry’s influence. Strategies should include components that shift demand for IT and denormalize its presence in communities, particularly in lower socioeconomic areas with higher smoking prevalence.

## Introduction

The United Kingdom has a strong history of developing and implementing strategies to tackle illicit tobacco. Following a sharp rise in the illicit tobacco market in the late 20th century, illicit cigarettes were estimated to comprise more than a fifth of the market in 2000/2001 and illicit hand-rolled tobacco comprised 61%.^1^ In the 21st century, a series of comprehensive cross-governmental measures to address illicit tobacco reduced the total and relative size of the illicit tobacco market.^2–7^ However, the illicit tobacco tax gap (the difference between tax owed on cigarettes and hand-rolled tobacco, and actual tobacco tax take) was still estimated to be 17.7% in 2021/2022.^8^ Around 10% of smokers reported using illicit tobacco in 2022 in England, with those from lower socioeconomic groups reporting higher rates of use.^9^

In October 2023, the Government announced a policy to raise the age of sale by 1 year every year. This has been welcomed by UK public health experts^10^ but its success is significantly dependent on minimizing the demand and supply of illicit tobacco, in addition to tackling underage sales. The policy included the commitment to an additional £30 million a year for enforcement agencies over a 5-year period, a total of £150 million, £100 million of which has been allocated to HM Revenue & Customs (HMRC) and Border Force, and £50 million to Trading Standards.^11^ The Government followed this in 2024 by publishing its new strategy to tackle illicit tobacco, “Stubbing out the problem.”^12^ HMRC has committed to establish a multiagency illicit tobacco taskforce, bringing together colleagues from HMRC, Border Force, and Trading Standards into a single team that collaborates closely with other law enforcement and intelligence partners.

It will be important for enforcement and public health agencies to understand the public’s exposure to and opinions toward illicit tobacco to guide policy implementation. We sought to characterize current attitudes toward and exposure to illicit tobacco in England.

## Methods

Supplementary questions on illicit tobacco were added to the England April 2022 wave of the monthly Smoking Toolkit Study (STS), a representative survey of adults aged 16 and over. The total sample size was 1767 adults, of which 262 were current smokers. The methodology for the STS is set out elsewhere.^13^ Participants were provided with a definition and examples of illicit tobacco and those who currently smoked tobacco were asked “Approximately when, if ever, did you last come across counterfeit or smuggled tobacco?” All participants were asked eight questions to elicit attitudes toward and knowledge of illicit tobacco. Missing data were excluded. Odds ratios (ORs) and confidence intervals (CIs) were calculated for the main analysis. Logistic regression was conducted for participant agreement with statements on illicit tobacco by demographic status, reported in Table S1.

## Results

Two hundred sixty-two current smokers were asked when they last came across illicit tobacco (Table 1). Data were missing for seven adults. Nearly one in five reported being exposed to illicit tobacco in the past year (18.3%, 95% CI 13.8% to 23.6%). Over a third of participants reported ever being exposed to illicit tobacco (35.1%, 95% CI 29.3% to 41.2%). Men had four times greater odds of coming across illicit tobacco than women in the last 12 months (OR 3.96, 95% CI 1.45 to 10.79). Those aged 55 and older had less than half the odds of coming across illicit tobacco in the last 12 months as those aged 18–34 (OR 0.44, 95% CI 0.15 to 1.29).

**Table 1.** Last Exposure to Illicit Tobacco by Participant Characteristics

		<12 months (%)	1–2 years (%)	>2 years (%)	Never (%)	Don’t know/can’t remember (%)	Refused (%)	Total (%)
**Subgroup**	**Characteristic**							
Total sample		48 (18.3)	10 (3.8)	34 (13.0)	148 (56.5)	15 (5.7)	7 (2.7)	262 (100.0)
Tobacco use	Cigarette smokers	39 (17.2)	10 (4.4)	31 (13.7)	128 (56.4)	13 (5.7)	6 (2.6)	227 (100.0)
	Other tobacco	9 (25.7)	3 (8.6)	0 (0.0)	20 (57.1)	2 (5.7)	1 (2.9)	35 (100.0)
Sex	Men	37 (25.0)	8 (5.4)	19 (12.8)	74 (50.0)	8 (5.4)	2 (1.4)	148 (100.0)
	Women	11 (10.0)	2 (1.8)	15 (13.6)	70 (63.6)	7 (6.4)	5 (4.5)	110 (100.0)
	Other	0 (0.0)	0 (0.0)	0 (0.0)	4 (100.0)	0 (0.0)	0 (0.0)	4 (100.0)
Age	18–34	23 (22.5)	5 (4.9)	10 (9.8)	60 (58.8)	2 (2.0)	2 (2.0)	102 (100.0)
	35–54	20 (22.0)	4 (4.4)	13 (14.3)	42 (46.2)	9 (9.9)	3 (3.3)	91 (100.0)
	55+	5 (7.4)	1 (1.5)	11 (16.2)	45 (66.2)	4 (5.9)	2 (2.9)	68 (100.0)
	Unknown	0 (0.0)	0 (0.0)	0 (0.0)	1 (100.0)	0(0.0)	0 (0.0)	1 (100.0)
Social grade	AB	7 (22.6)	0 (0.0)	3 (9.7)	17 (54.8)	2 (6.5)	2 (6.5)	31 (100.0)
	C1	14 (17.3)	5 (6.2)	11 (13.6)	42 (51.9)	8 (9.9)	1 (1.2)	81 (100.0)
	C2	7 (12.7)	3 (5.5)	8 (14.5)	35 (63.6)	1(1.8)	1 (1.8)	55 (100.0)
	D	8 (20.5)	0 (0.0)	5 (12.8)	23 (59.0)	1 (2.6)	2 (5.1)	39 (100.0)
	E	9 (24.3)	1 (2.7)	4 (10.8)	20 (54.1)	2 (5.4)	1 (2.7)	37 (100.0)
Ethnicity	White	41(19.2)	7 (3.3)	27 (12.6)	121 (56.5)	13 (6.1)	5 (2.3)	214 (100.0)
	Other ethnicity	7 (17.1)	3 (7.3)	5 (12.2)	25 (61.0)	0 (0.0)	1 (2.4)	41 (100.0)

A total of 1767 adults responded to questions on attitudes toward and knowledge of illicit tobacco (Table 2). There were no missing data. Two-thirds of respondents disagreed that selling illicit tobacco does not do any harm (67.1%, 95% CI 64.9% to 69.3%) and two-thirds agreed that illicit tobacco was a danger to children due to easy, cheap access (67.0%, 95% CI 64.8% to 68.2%). 58.2% (95% CI 55.9% to 60.4%) of respondents were not aware of or not confident of the tax losses due to illicit tobacco.

**Table 2.** Participant Attitudes Toward and Knowledge of Illicit Tobacco

Statement	Agree (%)	Neither agree nor disagree (%)	Disagree (%)	Don’t know (%)
Selling them doesn’t do anyone any harm	234 (13)	234 (13)	1,186 (67)	113 (6)
They are a danger to kids because they can buy them easily and cheaply	1,184 (67)	149 (8)	185 (10)	150 (8)
Buying them is no big deal	344 (19)	228 (13)	938 (53)	158 (9)
Over a billion pounds a year of tax is lost in the United Kingdom because of illegal cigarettes and tobacco	738 (42)	300 (17)	144 (8)	486 (28)
They bring crime into the local community	779 (44)	231 (13)	447 (25)	211 (12)
They encourage anti-social behavior	653 (37)	247 (14)	567 (32)	201 (11)
Illegal tobacco is associated with organized crime	1,019 (58)	174 (10)	257 (15)	218 (12)
Most smokers in my local area buy illegal tobacco	190 (11)	253 (14)	496 (28)	729 (41)

Those in the oldest age group had significantly greater odds of agreeing with all five statements that described the harms of illicit tobacco compared to those in the youngest age group (Table S1). This was also found for those who did not smoke compared to those who smoked. Those in the lowest socioeconomic group had greater odds of agreeing that buying illicit tobacco was “no big deal” (OR 1.77, 95% CI 1.15 to 2.26) and that most smokers in their local area buy illicit tobacco (OR 2.64, 95% CI 1.53 to 4.55). However, the lowest and highest socioeconomic groups had similar odds of agreeing with the five statements setting out the harm of tobacco.

## Discussion

The availability of illicit tobacco undermines the effectiveness of measures to reduce smoking prevalence by providing a cheaper alternative to UK duty-paid tobacco. Our analysis suggests that a significant portion of tobacco users in England, particularly younger men, have recently come across sources of illicit tobacco. Relatedly, the English public agree with the major arguments for taking greater action on illicit tobacco, most notably on cheap access for children, known to be price sensitive to tobacco.^14^

There are differences across socioeconomic groups, with those from lower socioeconomic backgrounds less likely to agree that selling illicit tobacco causes harm or that buying illicit tobacco is a major problem. This suggests that careful planning will be required for public health professionals and enforcement agencies to secure the support and trust of those living in lower-income areas in tackling illicit tobacco, where smoking prevalence is typically higher.^15^ There are examples of programs involving collaboration between health and enforcement partners in the United Kingdom. The North of England illicit tobacco program successfully changed public views on illicit tobacco and led to increased reporting by implementing a regional demand reduction communications program, backed up by enforcement at the local level.^16^ A significant proportion of the new funding linked to the illicit tobacco strategy should be devolved to such regionally led activity working with local authority partners, who operate both Trading Standards and public health departments, and have deep connections with their local communities. Specific funding arrangements should be made transparent to support evaluation and scrutiny of strategy delivery.

Study limitations include the single-instance cross-sectional design, which does not allow trends over time to be reported, and the relatively long periods of recall for past exposure to illicit tobacco, which may lack accuracy. Self-reporting of exposure to and views of illicit activity may also risk under-reporting.

The tobacco industry has been documented to be systematically seeking to control efforts to tackle illicit tobacco for the industry’s benefit, including tobacco tracking and tracing systems.^17^ The new illicit tobacco strategy and associated plans to change public attitudes must remain completely independent from the tobacco industry, as set out in the guidelines to Article 5.3 of the WHO Framework Convention on Tobacco Control and the Protocol to Eliminate Illicit Trade in Tobacco Products.^18,19^

## Supplementary material

Supplementary material is available at *Nicotine and Tobacco Research* online.

ntae118_suppl_Supplementary_Materials

## Data Availability

The data underlying this article are provided by The Smoking Toolkit Study by permission and are available at Open Science Framework. doi.org/10.17605/OSF.IO/W7CYB
