# Supplementary material for: Attitudes and Exposure to Illicit Tobacco in England, 2022
Source: Nicotine Tob Res. 2024 May 16;26(11):1591–4. doi: 10.1093/ntr/ntae118 (PMC11494475; doi:10.1093/ntr/ntae118)
Supplement: ntae118_suppl_Supplementary_Materials [file ntae118_suppl_supplementary_materials.docx]

**Supplementary material**

*Supplementary table 1:* *Univariate logistic regression of participant agreement with statements on illicit tobacco by demographic status*

|  |  | **Selling them doesn’t do anyone any harm** | **They are a danger to kids because they can buy them easily and cheaply** | **Buying them is no big deal** | **Over £1billion a year of tax is lost in the UK because of illegal tobacco** | **They bring crime into the local community** | **They encourage anti-social behaviour** | **Illegal tobacco is associated with organised crime** | **Most smokers in my local area buy illegal tobacco** |
| --- | --- | --- | --- | --- | --- | --- | --- | --- | --- |
|  |  | **Odds ratio (standard error)** | | | | | | | |
| Socio-economic group (ref: AB, highest) | C1 | 0.915 (0.236) | 1.153 (0.174) | 1.190 (0.217) | 0.954 (0.131) | 0.893 (0.121) | 1.064 (0.146) | 1.040 (0.146) | 1.174 (0.281) |
|  | C2 | 1.823** (0.488) | 1.069 (0.190) | 1.630** (0.332) | 1.428** (0.230) | 0.810 (0.130) | 0.755* (0.125) | 0.992 (0.165) | 1.767** (0.459) |
|  | D | 1.948** (0.596) | 0.837 (0.172) | 1.814** (0.423) | 1.091 (0.209) | 0.551*** (0.108) | 0.860 (0.168) | 0.750 (0.145) | 1.736* (0.521) |
|  | E | 1.396 (0.455) | 0.987 (0.207) | 1.770** (0.415) | 1.161 (0.223) | 0.839 (0.161) | 0.738 (0.147) | 1.069 (0.213) | 2.635*** (0.734) |
| Age (ref: 18 - 34) | 35 - 54 | 0.558*** (0.122) | 0.885 (0.123) | 0.451*** (0.068) | 1.879*** (0.247) | 2.445*** (0.325) | 1.185 (0.158) | 2.327*** (0.302) | 0.732 (0.143) |
|  | 55+ | 0.684* (0.137) | 1.038 (0.140) | 0.398*** (0.058) | 2.029*** (0.256) | 2.882*** (0.370) | 1.716*** (0.217) | 2.866*** (0.362) | 0.748 (0.139) |
| Smoking status (ref: non-smoker) | Smoker | 1.461* (0.314) | 0.945 (0.140) | 2.422*** (0.357) | 1.242 (0.168) | 0.718** (0.0985) | 0.384*** (0.0610) | 0.622*** (0.084) | 1.099 (0.228) |

*= p<0.10, ** = p<0.05, *** = p<.001
